# Supplementary material for: Fatigue, Depression and Health-Related Quality of Life in Patients with Post-Myocardial Infarction during the COVID-19 Pandemic: Results from the Augsburg Myocardial Infarction Registry
Source: J Clin Med. 2023 Oct 3;12(19):6349. doi: 10.3390/jcm12196349 (PMC10573677; doi:10.3390/jcm12196349)
Supplement: Supplementary file 1 [file jcm-12-06349-s001.zip › jcm-2626323-supplementary.pdf]

Supplementary material

Table S1: Results of the multivariable linear regression models. Associations between fatigue and depression and HRQoL (MacNew total score, and physical, emotional, and social dimensions) in men

	ß-estimate (95% CI)	p-value	R^2
**Physical dimension**			
Fatigue	-1.12 (-1.31 - -0.92)	<0.0001	0.3621
Depression	-1.72 (-1.98 - -1.46)	<0.0001	0.4102
No depression/no fatigue	Ref.		0.5049
No depression/fatigue	-0.83 (-1.02 - -0.64)	<0.0001	
Depression/no fatigue	-2.12 (-2.81 - -1.42)	<0.0001	
Depression and fatigue	-2.03 (-2.30 - -1.77)	<0.0001	
**Emotional dimension**			
Fatigue	-1.15 (-1.33 - -0.96)	<0.0001	0.3371
Depression	-1.91 (-2.15 - -1.68)	<0.0001	0.4489
No depression/no fatigue	Ref.		0.5445
No depression/fatigue	-0.77 (-0.94—0.60)	<0.0001	
Depression/no fatigue	-1.89 (-2.52—1.27)	<0.0001	
Depression and fatigue	-2.25 (-2.48—2.01)	<0.0001	
**Social dimension**			
Fatigue	-1.08 (-1.27 - -0.88)	<0.0001	0.3376
Depression	-1.77 (-2.02 - -1.52)	<0.0001	0.4224
No depression/no fatigue	Ref.		0.5095
No depression/fatigue	-0.77 (-0.95 - -0.59)	<0.0001	
Depression/no fatigue	-2.23 (-2.90 - -1.55)	<0.0001	
Depression and fatigue	-2.05 (-2.30 - -1.80)	<0.0001	
**Total score**			
Fatigue	-1.08 (-1.26 - -0.90)	<0.0001	0.3597
Depression	-1.76 (-1.99 - -1.53)	<0.0001	0.4504
No depression/no fatigue	Ref.		0.5468
No depression/fatigue	-0.76 (-0.93 - -0.60)	<0.0001	
Depression/no fatigue	-2.04 (-2.65 - -1.43)	<0.0001	
Depression and fatigue	-2.06 (-2.29 - -1.83)	<0.0001	

Adjusted for age, diabetes, prior stroke, German nationality, highest school education, living alone, smoking status, BMI, any recanalisation therapy, hypertension, STEMI infarction, employment status, prior SARS-CoV-2 infection

Table S2: Results of the multivariable linear regression models. Associations between fatigue and depression and HRQoL (MacNew total score, and physical, emotional, and social dimensions) in women

	ß-estimate (95% CI)	p-value	R^2
**Physical dimension**			
Fatigue	-1.43 (-1.74 - -1.12)	<0.0001	0.4703
Depression	-1.41 (-1.84 - -0.98)	<0.0001	0.3432
No depression/no fatigue	Ref.		0.5246
No depression/fatigue	-1.16 (-1.49 - -0.84)	<0.0001	
Depression/no fatigue*	-	-	
Depression and fatigue	-1.96 (-2.36 - -1.56)	<0.0001	
**Emotional dimension**			
Fatigue	-1.41 (-1.73 - -1.10)	<0.0001	0.4540
Depression	-1.84 (-2.23 - -1.45)	<0.0001	0.4788
No depression/no fatigue	Ref.		0.5979
No depression/fatigue	-0.96 (-1.27 - -0.66)	<0.0001	
Depression/no fatigue*	-	-	
Depression and fatigue	-2.29 (-2.66 - -1.92)	<0.0001	
**Social dimension**			
Fatigue	-1.32 (-1.65 - -0.99)	<0.0001	0.4143
Depression	-1.45 (-1.88 - -1.02)	<0.0001	0.3528
No depression/no fatigue	Ref.		0.4846
No depression/fatigue	-1.00 (-1.35 - -0.66)	<0.0001	
Depression/no fatigue*	-	-	
Depression and fatigue	-1.92 (-2.34 - -1.50)	<0.0001	
**Total score**			
Fatigue	-1.36 (-1.65 - -1.07)	<0.0001	0.4726
Depression	-1.56 (-1.94 - -1.18)	<0.0001	0.4183
No depression/no fatigue	Ref.		0.5744
No depression/fatigue	-1.01 (-1.30 - -0.72)	<0.0001	
Depression/no fatigue*	-	-	
Depression and fatigue	-2.03 (-2.38 - -1.68)	<0.0001	

Adjusted for age, diabetes, prior stroke, German nationality, highest school education, living alone, smoking status, BMI, any recanalisation therapy, hypertension, STEMI infarction, employment status, prior SARS-CoV-2 infection

*There were no women in this group

Table S3: Results of the multivariable linear regression models. Associations between fatigue and depression and HRQoL (MacNew total score, and physical, emotional, and social dimensions) in AMI patients aged <70 years

	ß-estimate (95% CI)	p-value	R^2
**Physical dimension**			
Fatigue	-1.24 (-1.45 - -1.03)	<0.0001	0.4671
Depression	-1.49 (-1.78 - -1.20)	<0.0001	0.4120
No depression/no fatigue	Ref.		0.5370
No depression/fatigue	-0.92 (-1.14 - -0.69)	<0.0001	
Depression/no fatigue	-0.91 (-2.48 - 0.66)	0.2538	
Depression and fatigue	-1.83 (-2.10 - -1.56)	<0.0001	
**Emotional dimension**			
Fatigue	-1.37 (-1.59 - -1.15)	<0.0001	0.4462
Depression	-1.95 (-2.24 - -1.67)	<0.0001	0.4944
No depression/no fatigue	Ref.		0.5966
No depression/fatigue	-0.85 (-1.07 - -0.63)	<0.0001	
Depression/no fatigue	-0.73 (-2.27 - 0.81)	0.3510	
Depression and fatigue	-2.28 (-2.55 - -2.01)	<0.0001	
**Social dimension**			
Fatigue	-1.22 (-1.44 - -1.01)	<0.0001	0.4366
Depression	-1.55 (-1.84 - -1.26)	<0.0001	0.4098
No depression/no fatigue	Ref.		0.5209
No depression/fatigue	-0.86 (-1.09 - -0.64)	<0.0001	
Depression/no fatigue	-1.00 (-2.59 - 0.60)	0.2199	
Depression and fatigue	-1.87 (-2.15 - -1.59)	<0.0001	
**Total score**			
Fatigue	-1.25 (-1.45 - -1.05)	<0.0001	0.4725
Depression	-1.67 (-1.93 - -1.40)	<0.0001	0.4715
No depression/no fatigue	Ref.		0.5870
No depression/fatigue	-0.85 (-1.05 - -0.64)	<0.0001	
Depression/no fatigue	-0.94 (-2.37 - 0.49)	0.1963	
Depression and fatigue	-1.98 (-2.23 - -1.73)	<0.0001	

Adjusted for age, diabetes, prior stroke, German nationality, highest school education, living alone, smoking status, BMI, any recanalisation therapy, hypertension, STEMI infarction, employment status, prior SARS-CoV-2 infection

Table S4: Results of the multivariable linear regression models. Associations between fatigue and depression and HRQoL (MacNew total score, and physical, emotional, and social dimensions) in AMI patients aged ≥70 years

	ß-estimate (95% CI)	p-value	R^2
**Physical dimension**			
Fatigue	-1.06 (-1.32 - -0.81)	<0.0001	0.3383
Depression	-1.70 (-2.02 - -1.37)	<0.0001	0.4089
No depression/no fatigue	Ref.		0.4984
No depression/fatigue	-0.84 (-1.09 - -0.60)	<0.0001	
Depression/no fatigue	-2.42 (-3.24 - -1.60)	<0.0001	
Depression and fatigue	-2.07 (-2.41 - -1.73)	<0.0001	
**Emotional dimension**			
Fatigue	-1.02 (-1.26 - -0.79)	<0.0001	0.3211
Depression	-1.83 (-2.11 - -1.55)	<0.0001	0.4686
No depression/no fatigue	Ref.		0.5487
No depression/fatigue	-0.73 (-0.94 - -0.52)	<0.0001	
Depression/no fatigue	-2.12 (-2.82 - -1.42)	<0.0001	
Depression and fatigue	-2.20 (-2.49 - -1.91)	<0.0001	
**Social dimension**			
Fatigue	-0.97 (-1.23 - -0.72)	<0.0001	0.3085
Depression	-1.72 (-2.04 - -1.41)	<0.0001	0.4202
No depression/no fatigue	Ref.		0.4949
No depression/fatigue	-0.74 (-0-98 - -0.50)	<0.0001	
Depression/no fatigue	-2.48 (-3.28 - -1.69)	<0.0001	
Depression and fatigue	-2.03 (-2.36 - -1.70)	<0.0001	
**Total score**			
Fatigue	-0.99 (-1.21 - -0.76)	<0.0001	0.3365
Depression	-1.70 (-1.98 - -1.42)	<0.0001	0.4252
No depression/no fatigue	Ref.		0.5438
No depression/fatigue	-0.74 (-0.95 - -0.54)	<0.0001	
Depression/no fatigue	-2.28 (-2.98 - -1.59)	<0.0001	
Depression and fatigue	-2.04 (-2.32 - -1.75)	<0.0001	

Adjusted for age, diabetes, prior stroke, German nationality, highest school education, living alone, smoking status, BMI, any recanalisation therapy, hypertension, STEMI infarction, employment status, prior SARS-CoV-2 infection
